# HIV positive sero-status disclosure and its determinants among people living with HIV /AIDS following ART clinic in Jimma University Specialized Hospital, Southwest Ethiopia: a facility- based cross-sectional study

**DOI:** 10.1186/s13690-017-0251-3

**Published:** 2018-01-15

**Authors:** Tamiru Tesfaye, Jiregna Darega, Tefera Belachew, Abebe Abera

**Affiliations:** 1 Department of Nursing, Goba Referral Hospital, Madda Walabu University, Bale-Robe, Ethiopia; 2 Department of Public Health, College of Medicine and Health Science, Ambo University, West Shewa, Ethiopia; 3 Department of Human Nutrition, College of Public Health and Medical Sciences, Jimma University, Jimma, Ethiopia; 4 Department of Nursing, College of Public Health and Medical Sciences, Jimma University, Jimma, Ethiopia

**Keywords:** ART patients, associated factors, Disclosure, Jimma University Specialized Hospital, and People living with HIV/AIDS

## Abstract

**Background:**

Even though, the disclosure of HIV sero- status to sexual partners, friends or relatives is the main tool for prevention and care strategies, most of the HIV/AIDS patients do not inform their close friends. The most common reasons for not disclosure of their status to the community were majorly fear of social rejection and discriminations. Therefore, this study assessed the HIV positive sero-status disclosure and its determinants among People Living with HIV /AIDS (PLWH/A) followed by the Antiretroviral therapy (ART) Clinic in Jimma University Specialized Hospital, Southwest Ethiopia.

**Methods:**

A facility based cross-sectional study design was used among 351 ART patients that selected by systematic random sampling from ART clinic of Jimma University Specialized Hospital in March-2014. Data were collected through interviewer-administered questionnaires and analyzed using SPSS version 20.0 software. In a descriptive analysis frequency, mean and percentage were calculated. Bivariate and multivariate analyses were used to identify associated factors and the association between the explanatory and dependent variables was estimated.

**Results:**

Only 37.6% (*n* = 132) were revealed their HIV positive status to anyone. Disclosure was done towards the sexual partners (88.6%), close family (72.7%) and a larger population (18.2%). Age ≤ 39 years (AOR = 0.014 [95%, CI = 0.005, 0.037]),Male sex (AOR = 3.039, [95% CI = 1.164, 7.935]), WHO stage III – IV at ART start(AOR = 2.766, [95%, CI = 1.321, 5.791]), presence of comorbidity (AOR = 2.500, [95%, CI = 1.483, 4.214]), having any clinical symptoms for HIV(AOR = 2.98, [95%, CI = 1.724, 5.152]),Low physical domain related quality of life (AOR = 3.83, [95%, CI = 2.008, 7.315]) and high social domain related quality of life (AOR = 0.053, [95%, CI = 0.022, 0.125]) were statistically significant association with their HIV sero-status disclosure.

**Conclusions:**

Findings of this study indicated, the disclosure of HIV status is very low. Discloser is more likely when the patient is older, male, and has a higher level of education. Clinical determinants for disclosure was the WHO stage III-IV, treatment duration of ≥2 years, comorbidity, presence of clinical symptoms for HIV, low physical domain related quality of life, low social domain related quality of life and low overall quality of life.

## Background

HIV is referred to (Human Immunodeficiency Infection) which is the most dangerous virus which is the major cause of AIDS in human life. It continued to spread worldwide and one of serious health challenges. Although much of the news on AIDS is encouraging, the challenges become continued [1].

Globally, there were 36.7 million people living with HIV in 2015. This was high of 33.3 million in 2010. These increments in a number of patients were resulted because of continuing new infections, people living longer with HIV, and general population growth. 1.1 million People died of AIDS in 2015. There were about 2.1 million new infections in 2015 or about 5700 new infections per day. An estimated 32.1 million of adults aged 15–49 years worldwide are living with HIV [2]. 1.8 million Children living with HIV, 110,000 AIDS-related deaths, and 150,000 new infections among children in 2015 [3].

Sub-Saharan Africa, the hardest hit region, is home to nearly 70% of people living with HIV but only about 13% of the world’s population [4]. The sub-region of Eastern and Southern Africa were home to more than half (52%) of all people living with HIV, as well as more than half of the children living with HIV (56%) [5].

Ethiopia has a large and very vulnerable populations that there were more than half million (671,941) HIV positive population, 14,405 new HIV infections and 24,813 deaths in 2016 [6].

Disclosure of human immune deficiency virus (HIV) positive status has a key role in the prevention and control of HIV/AIDS. The failure of people infected with HIV to disclose their positive status can expose their sexual partners and other relatives that have close contact with them to the virus [7]. In developing countries, the rates of disclosure of HIV sero- status was very low compared to developed countries. The study showed that the rates ranged from 16.7% to 86% with the average of 49% that was almost half of the HIV positive patients not exposed their sero-status to other people including their sexual partners. Whereas, in average 79% of HIV positive patients were disclosed their HIV sero-status to other peoples’ in study done in developed countries [8]. Likewise, in Ethiopia, very low percentage of HIV positive patients disclosed their sero- status to other people including their sexual partners.

There are different factors that affect the disclosure of their HIV status; such as Marital status, knowledge of partner HIV status, fearing negative outcomes of disclosure, their communication skills, initiation of anti-retroviral, receiving ongoing counseling and duration of HIV related care follow up are some of the identified reasons [9, 10]. The knowledge of those factors is cored for prevention and control of HIV/AIDS strategies. Therefore, this study assessed the prevalence of HIV positive sero - status disclosure and its determinants among People Living with HIV /AIDS following ART Clinic in Jimma University Specialized Hospital, Southwest Ethiopia.

## Methods

### Study setting and participants

Facility (Hospital) based cross- sectional study design was conducted in Jimma University Specialized Hospital ART clinic from March 1 to 30, 2014. Jimma University Specialized Hospital (JUSH) is one the oldest public hospital in the country. It was established in 1937 by Italian invaders for the service of their soldiers. Geographically, it is located in Jimma city that is located at 357 km from Addis Ababa [11]. JUSH is the teaching and referral hospital that have around 450 beds and more than 750 staffs of both supportive and professionals. It provides services approximately for 9000 inpatients and 80,000 outpatients’ attendants in a year. JUSH ART clinic pioneered the use of antiretroviral drugs in Ethiopia in 2002. The clinic currently gives follow up service for about 6260 HIV/AIDS patients. On average, about130 patients were visiting the clinic on a day. The appointments follow up given to patients in every one to two months. The clinic staffed with internist, the senior health officer and nurses who were trained in specific HIV/AIDS disease patient cares [12].

### Sample size determinations and Sampling techniques

The sample was determined using single population proportion formula using EPI Info 7.1.0.6 version was applied with an assumption of a level of confidence of the study 95%, sampling error tolerated 5%, Proportion of disclosure to relatives 33.2% in the same institution (Jimma University Specialized Hospital) at 2007 [13]. Finally, 358 sample sizes were obtained.

Study participants were selected by using systematic random sampling technique. There were a total of 6260 patients registered for ART care in the hospital. Per day, there was a high patient flow; approximately 130 patients attend the PLWHA clinic each day. To finish data collection within one month, every day around 18 study participants were recruited. Therefore, within the interval of the 7th or every 7th patient attending the clinic were involved in an interview until the required sample of 358 was recruited. As patients attended the clinic on a monthly basis and above, no patients included twice during the recruitment period. The study includes adult patients above the age of eighteen years old due to ethical issues.

### Data collection Instruments and methods

The questioners were adapted from related kinds of literature [13–18]. The questioners assessed mainly socio - demographic factors like age, educational status, occupation, marital status, etc.; disease related care factors like CD4 count, WHO stage, the length of treatment, general health conditions, etc. and social relationship related factors such as reminder of drugs, with whom they live, self-care status, Data were collected both by Afan Oromo and Amharic questionnaires. Five BSc nurses working in JUSH ART clinic who could speak both Amharic and Afan Oromo were collected the data by face-to-face interview. One BSc Nurse who works in JUSH ART clinic was assigned as the supervisor at the time of data collections to supervise the overall data collection conditions including technical activities. The patients were interviewed after they got the service they required from the ART clinic.

### Data quality assurance

The questionnaires were pre-tested on 5% of the sample in Jimma town, Shanan Gibe Hospital on HIV positive patients that started HAART drug treatment at the ART clinic before the actual data collections and amendments were made accordingly. Language experts translated the questioners into two local languages (Amharic and Afan Oromo) and then back to English to make sure the consistency of the questionnaires, then correction was done accordingly. The data collectors and supervisors were trained on data collection tools and procedures for one day. On top of this, supervisors were followed data collectors. Both supervisors and investigators were also checked for the collected data clear and completeness in every day of a period of data collections.

### Data Processing and analysis

Before data entry, questionnaires were checked for its completeness. Data was entered into Epidata version 3.0 and then exported to SPSS version 20.0 for further analysis. The descriptive analysis like percentage, frequency and mean were calculated. The bivariate logistic regression analysis was used to identify associations between variables. The variables that had p – value ≤0.3, have association in other similar research and variables that suspected to have association study variables were transferred to multiple logistic regression models. The possible effects of confounders were controlled through multivariate logistic regression analysis. The association between the explanatory and dependent variables were assessed at the *p*-value of 0.05. The variables that shown p - value <0.05 were considered as statistically significant variables in multiple logistic regression model. The degree of association between independent and dependent variables were assessed using adjusted odds ratio with 95% confidence interval. The findings were presented in the form of narratives, tables, pie charts, and graphs.

## Results

### Socio-demographic Characteristics

From the total 358 sampled and 351 of the study participants participated in the study that result in 98.1% of response rate. From total of study participants, 228 (65%) of them were females. Most of the participants, 237 (67.5%) aged less than 40 years with the mean age of 34.9 (SD ± 8.68) (min 20 years and max 67 years old). Never married were dominant that accounts 152 (43.3%), then followed by married 99 (28.2%) and divorced 69 (19.7%). The majority, 143 (40.7%) were attended primary school educations and followed by secondary school 104 (30.5%). Oromo accounts 153 (42.7%) by ethnicity and Muslims 102 (29%) by religion. Whereas, 247 (70.4%) of them were currently employed [Table 1].

**Table 1:** Socio demographic characteristics of PLWHA on follow up in Jimma University Specialized hospital in ART clinic, southwest Ethiopia, 2014 (*N* = 351)

Variables	Frequency	Percentage (%)
Age of the respondent		
1. ≤ 39 years	237	67.5
2. ≥ 40 years	114	32.5
Sex of Sex of respondents respondents		
1. Male	123	35
2. Female	228	65
Marital status		
1. Single	152	43.3
2. Married	99	28.2
3. Widowed	31	8.8
4. Divorce	69	19.7
Educational level		
1. No formal education	72	20.5
2. Grade: 1–8	141	40.2
3. Grade: 9–10	107	30.5
4. 10+, certificate and above	31	8.8
Ethnicity		
1. Oromo	153	43.6
2. Amhara	96	27.4
3. Tigre	18	5.1
4. Dawuro	34	9.7
5. Yem	36	10.3
6. Other*	14	4
Religion		
1. Muslim	102	29.1
2. Orthodox	181	51.6
3. Protestant	68	19.3
Occupation		
1. Currently Employed	239	68.1
2. Currently not employed	112	31.9
Income of respondents		
1. ≤ 500 ETB	253	72.1
2. 501–1000 ETB	48	13.7
3. ≥ 1001 ETB	50	14.2

### Clinical conditions of Participants

The majority, 248 (70.7%) had CD4 count less than <250 cell/ml when they started ART follow up services. About, 278 (79.2%) of the respondents had ≥250 cells/ml CD4 count level. Three- forth, 283 (80.6%) of the them were on stage I - II of WHO classifications. Large number, 233 (66.4%) of them was taking prophylaxes. Moreover, 247 (70.4%) of respondents were found in good general health conditions currently. Around, 273 (77.8%) of them were present on better life currently than last year [Table 2].

**Table 2:** Clinical conditions of PLWHA on follow up in Jimma University Specialized hospital in ART clinic, southwest Ethiopia, 2014(*N* = 351)

Variables	Alternatives	Frequency	Percentage
CD4 count when ART started	1. < 250 cell/ml	248	70.7
	2. ≥ 250 cell/ml	103	29.3
CD4 count currently	1. < 250 cell/ml	73	20.8
	2. ≥ 250 cell/ml	278	79.2
WHO stage at start	1. Stage I - II	286	81.5
	2. Stage III - V	65	18.5
Current WHO stage of HIV	1. Stage I - II	283	80.6
	2. Stage III- V	68	19.4
Vests clinic for health seeking	1. Yes	285	81.2
	2. No	66	18.8
Discontinuation of OI prophylaxes	1. Yes	233	66.4
	2. No	118	33.6
How long they stayed on treatment	1. < 2 years	143	40.7
	2. ≥ 2 years	208	59.3
General health condition	1. Good	247	70.4
	2. Poor	104	29.6
1.1.Faced body pain in last 4 weeks	1. Not faced	217	61.8
	2. mild pain	103	29.3
	3. severe pain	31	8.8
Healthy as any body	1. Yes	223	63.5
	2. No	128	36.5
Compared to last year how they rate their health status	1. better now than a year	273	77.8
	2. worse now than a year	78	22.2
Can perform any physical exercise	1. yes	215	61.3
	2. No	136	38.7
Have attended health education	1. Yes	287	81.8
	2. No	64	18.2

### HIV-positive status disclosure

**Fig. 1:**
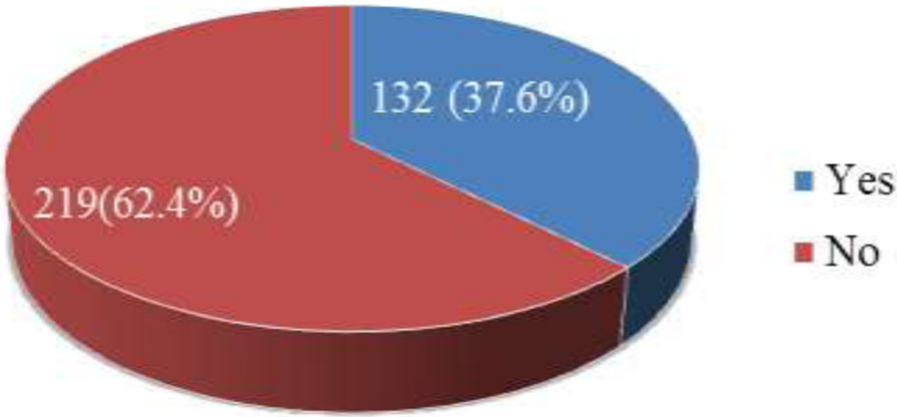
The HIV Disclosure status of PLWHA on follow up in Jimma University Specialized hospital in ART clinic, southwest Ethiopia, 2014

**Fig. 2:**
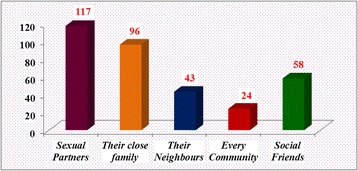
The HIV Disclosure status to others peoples among PLWHA on follow up in Jimma University Specialized hospital in ART clinic, southwest Ethiopia, 2014

Of the respondents, only 132 (37.6%) were disclosed as they were HIV sero -status positive patients [Fig. 1). Out of these disclosed patients, almost all of them 117 (88.6%) exposed to their sexual partner followed by their close families 96 (72.7%). But, only 24 (18.2%) of them disclosed to any people. [Fig. 2].

### Socio demographic, social and behavioral factors that associated with HIV positive status disclosure among participants

In bivariate analysis; age, sex, educational status, marital status, relationship with other people, drinking alcohol, perceived HIV related stigma, a social domain of quality of life and Substance abusing behaviors of participants had significant association with HIV sero-status disclosure.. But in multivariate analysis; age, sex, educational status and the social domain of quality of life were significantly associated with HIV sero-status disclosure.

Elaborately, participants those aged ≤39 years were less likely to disclose (AOR = 0.014 [95%, CI = 0.005, 0.037]) than those aged ≥40 years. Similarly, males were three times more likely to disclose their HIV sero - status than females (AOR = 3.039, [95% CI = 1.164, 7.935]). Again, secondary school (AOR = 4.368, [95%, CI = 1.450, 13.153]) and ≥10+ and above attendants (AOR = 26.291, [95%, CI = 3.886, 7.876]) were more likely to disclose their HIV sero -status to other persons than the rest. Moreover, those had high level of social domain of quality of life were less likely to disclose than those had low level of social domain of quality of life (AOR = 0.053, [95%, CI = 0.022, 0.125]) [Table 3].

**Table 3:** Associations of Socio demographic, social and behavioral factors with HIV positive status disclosure of PLWHA on follow up in Jimma University Specialized hospital in ART clinic, southwest Ethiopia, 2014

Independent Variables	Alternatives	HIV status disclosure		COR [95% C.I]	*P*- Value		AOR [95% C.I]
		Yes	No		COR	AOR	
Age of respondents	≤ 39 years	31	206	0.019 (0.010–0.039)	.000	.000	0.014(0.005–0.037)
	≥ 40 years	101	13	1.00			1.00
Sex of respondents	Male	71	52	3.536 (2.230–5.607)	.001	.023	3.039(1.164–7.935)
	Female	61	167	1.00			1.00
Marital Status	Single	39	113	1.00			1.00
	Married	29	70	8.330 (3.445–20.144)	.000	.554	1.338(.510–3.509)
	Divorce	41	28	6.940 (2.78–17.301)	.000	.097	.407(.141–1.175)
	Widowed	23	8	1.963 (0.769–5.012)	.158	.316	.441(.089–2.186)
Educational level of respondents	No formal education	29	41	1.00			1.00
	Grade 1–8	76	68	0.745 (.421–1.318)	.312	.899	1.066(.396–2.872)
	Grade 9–10	21	86	3.276 (1.683–6.377)	.000	.009	4.368(1.450–13.153)
	10+ and above	6	24	3.333 (1.220–9.106)	.019	.001	26.291(3.886–7.876)
Occupation of respondents	Currently employed	83	156	.684 (.432–1.082)	.105	.889	1.064(.442–2.563)
	Currently not employed	49	63	1.00			1.00
Average monthly income	≤ 500ETB	103	150	1.00			1.00
	501- 1000ETB	16	32	1.373 (.717–2.632)	.339	.320	1.974(.517–7.532)
	≥ 1001ETB	13	37	1.954 (.990–3.857)	.053	.476	1.531(.474–4.944)
Discontinuation of OI prophylaxes	No	84	149	0 .822(.522–1.295)	.398		
	Yes	48	70	1.00			1.00
Reminder while they took drugs	Self	106	169	1.206(.708–2.054)	.490		
	Other person	26	50	1.00			
Currently with whom they live	With another person	112	190	1.170(.632–2.165)	.617		
	Alone	20	29	1.00			
Relationship with other people	Friendly	104	132	2.297(1.402–3.761)	.001	.979	1.013(0.385–2.667)
	Discrimination	29	86	1.00			
Drinks alcohol	Yes	49	64	5.408 (2.219–13.178)	.000	.270	1.647(.679–3.994)
	No	83	155	1.00			
Do you smoke cigarette	yes	38	59	1.096 (.678–1.773)	.708		
	No	94	160	1.00			
Vests clinic for health seeking	Yes	107	178	0.986(.568–1.712)	.960		
	No	25	41	1.00			
Perceived HIV related stigma	High	65	164	1.00			1.00
	Low	64	52	3.105(1.950–4.945)	.000	.561	1.323(.515–3.396)
Social domain of quality of life	Low	40	183	1.00			1.00
	High	92	36	11.692(6.984–19.573)	.000	.000	0.053(0.022–0.125)
Substance abusing	Yes	94	113	2.320(1.464–3.678)	.000	.487	1.342(.586–3.073)
	No	38	106	1.00			1.00
Self-care status	Low	96	139	1.535(.958–2.459)	.075	.795	.883(0.348–2.246)
	High	36	80	1.00			1.00

### Clinical factors that associated with HIV positive status disclosure among participants

Those variables such as; performing of any physical exercise, CD4 count level when ART started, WHO stage at start of ART, general health conditions of the patients, how long they stayed on treatment, whether they perceive themselves as healthy as anybody or not, having comorbidity conditions of other diseases, having any clinical symptoms for HIV/AIDS, Feeling they had about their health conditions, how they rate their health status by comparing to last year, their status of physical domains of quality of life and their status of quality of life showed significant association with disclosure of HIV sero – status during bivariate analysis.

Then those variables that had p – values ≤0.3 were transferred to multiple logistic regression models. Accordingly, ability to perform of any physical exercise, WHO stage at start of ART, general health conditions, length of time they stayed on HAART treatment, having comorbidity conditions with other diseases, having any clinical symptoms of HIV/AIDS, status of physical domains of quality of life and overall quality of life had statistically significant association with disclosure of HIV positive sero – status.

For further explanation, participants those couldn’t perform any physical exercise were two times more likely to disclose their HIV sero – status (AOR =1.922, [95% CI = 1.142, 3.235]). In addition, those at stage III – IV were around triply more to disclose their HIV sero – status when compared to those at stage I- II (AOR = 2.766, [95%, CI = 1.321, 5.791]). Moreover, participants those had comorbidity with other diseases were 2.5 times more likely to disclose than those had no comorbidities (AOR = 2.500, [95%, CI = 1.483, 4.214]). Similarly, those had any clinical symptoms of HIV/AIDS were three times more to disclose than those hadn’t clinical symptoms (AOR = 2.98, [95%, CI = 1.724, 5.152]). Likewise, participants that present on low status of the physical domain of quality of life were four times higher to disclose than their opponents(AOR = 3.83, [95%, CI = 2.008, 7.315]). Finally, clients those present on the low general quality of life were twofold more likely to disclose than their counterparts[Table 4].

**Table 4:** Association of Clinical factors with HIV positive status disclosure of PLWHA on follows up in Jimma University Specialized hospital in ART clinic, southwest Ethiopia, 2014

Independent Variables	Alternatives	HIV status disclosure		COR [95% C.I]	*P*- Value		AOR [95% C.I]
		Yes	No		COR	AOR	
Perform any physical exercise	Yes	67	148	1.00			1.00
	No	65	71	2.022(1.298–3.150)	.002	.014	1.922(1.142–3.235)
CD4 count when ART started	< 250 cell/ml	82	91	2.192(1.410–3.409)	.000	.138	1.509(0.876–2.600)
	≥ 250 cell/ml	51	127	1.00			1.00
CD4 count currently	< 250 cell/ml	12	61	1.060(.657–1.711)	.811		
	≥ 250 cell/ml	82	196	1.00			
WHO stage at start	Stage I-II	119	167	2.850(1.486–5.469)	.002	.007	2.766(1.321–5.791)
	Stage III-IV	13	52	1.00			1.00
Current WHO stage of HIV	Stage I-II	107	178	0.986(.568–1.712)	.960		
	Stage III-IV	25	41	1.00			
General health condition	Good	82	165	1.00			1.00
	Poor	50	54	1.863 (1.168–2.972)	.009	.048	1.741(1.005–3.017)
How long they stayed on treatment	< 2 years	39	104	1.00			1.00
	≥ 2 years	93	115	2.157(1.364–3.411)	.001	.015	1.941(1.136–3.316)
They healthy as any body	Yes	99	158	1.00			1.00
	No	33	61	1.601(1.166–2.198)	.004	.600	1.185(0.628–2.237)
Have comorbidity conditions	Yes	75	62	3.332(2.119–5.240)	.000	.001	2.500(1.483–4.214)
	No	57	157	1.00			1.00
Have any Clinical symptoms for HIV	Yes	103	104	3.927(2.406–6.411)	.000	.000	2.980(1.724–5.152)
	No	29	115	1.00			1.00
Felt their health is excellent	Yes	86	166	1.00			1.00
	No	46	53	1.675(1.044–2.689)	.033	.853	0.945(0.520–1.717)
Felt sick earlier than other people	Yes	89	156	0.836(.524–1.333)	.452		
	No	43	63	1.00			
Compared to last year how they rate your health	Better now than a year	89	184	1.00			1.00
	Worse now than a year	43	35	2.540(1.521–4.242)	.000	.087	1.698(0.926–3.113)
Bathing and dressing	Not limited	82	155	1.00			
	Limited	50	64	1.477(.935–2.331)	.094	.313	1.537(0.666–3.546)
Physical domain of quality of life	Low	59	77	1.490(.959–2.317)	.076	.000	3.833(2.008–7.315)
	High	73	142	1.00			1.00
Psychological domain	Low	91	147	0.920(.578–1.46)	.724		
	High	41	72	1.00			
Quality of life of HIV/AIDS patients	Low	70	67	2.634(1.687–4.111)	.000	.000	4.349(2.301–8.219)
	High	62	152	1.00			1.00

## Discussions

This study had assessed the HIV positive sero-status disclosure and its determinants among People Living with HIV /AIDS following ART Clinic in Jimma University Specialized Hospital, Southwest Ethiopia. Accordingly, from the total respondents, only 132 (37.6%) were disclosed their HIV sero –status positivity in general. Out of these disclosed patients, almost all of them, 117 (88.6%) exposed to their sexual partner followed by their close families 96 (72.7%). But, only 24 (18.2%) of them publicized as they infected by the HIV/AIDS to every community.

In contrary to this study results, the study done in National Hospital Abuja, Nigeria showed that 228 (95.0%) had disclosed their HIV status and most, 121 (50.4%) had disclosed to their sexual partners [19]. In a similar way, the research done in North-Eastern Nigeria reflected that most 97.5% had disclosed their HIV status and majority 36.8%disclosed to their spouses [17]. Also the study done in Mwanza, Tanzania, 252 (93.3%) of participants disclosed their sero-status to other people [20]. Moreover, this study has in contrary result with the study done at Sokode Regional Hospital, Togo that 131 (60.9%) had disclosed their HIV sero-status to their sexual partners [21]. The above articles’ findings showed that there was higher disclosure rate to other peoples and low disclosure rate to their sexual partners. But, for this study finding, disclosure to sexual partners were higher than that the total people. This difference in findings might be resulted due to the sociocultural differences of the underlying communities.

Again, this study results were inconsistent with the research done in Mekelle Hospital, Ethiopia, in that the overall HIV status disclosure to sexual partner was 186 (57.4%) [7]. Moreover, the other study done in Mekelle Hospital, Ethiopia on women revealed that 201 (63.8%) of women were disclosed their HIV sero- status to their partners [22]. This discrepancy in the results of both studies might be resulted due to the time period and difference in socio- demography, cultures and socio - economies of the study participants.

In another way, this study results were almost conformed with the study done in Kampala, Uganda that 646 (81%) reportedas they had disclosed their HIV status to their sexual partners [23].

Again, the findings of this study similar to the study done in Hawassa University Referral Hospital, SNNPR, Ethiopia, which 85.7% of the women had disclosed their HIV positive status to their sexual partners [14]. Similar to the above study, the investigation done in Jimma Hospital, Ethiopia has shown that the majority (94.5%) disclosed their HIV sero- positive status result to at least one person and 90.8% disclosed to their current main partners [13]. The resulted similarity might be due to study place the closeness and the same with this study area.

Moreover, this study were in line with the study done in Kemissie district, Northeast Ethiopia that 335 (93.1%) had disclosed their HIV sero- positive results to their main current sexual partners, 263 (73.1%) had disclosed their HIV sero- status positivity results to at least one of their families and 219 (60.8%) had told their HIV sero- positive result to one or more of their friends [16]. Additionally, the research done in Axum Health Facilities, Tigray, Northern Ethiopia obtained that the majority (80.1%) disclosed their HIV sero - positive result to at least one person. From currently have a sexual partner, 81.2% disclosed to their current sexual partners [24]. Furthermore, a study done in Ambo Hospital, Ethiopia also revealed that the prevalence of HIV sero-positive status disclosure to at least one person was 86.2% and 84.9% study subjects were disclosed their HIV positive sero status to their sexual partners [25]. Those above listed studies had similar findings with this study. This similarity might be due to study period closeness to each other and underlying study participants had a much close sociocultural and economic background.

The practice of the disclosure of their HIV-positive status information to other peoples is not stress-free itself. They had terror or fear of the community reactions towards them after their disclosure. As a result, to make smooth the disclosure process for better consequences for individuals and their close groups, scanning of factors that contribute to the decision to disclose is very core points in supporting the actions of disclosure in multi dimensions. Accordingly, this investigation identified different factors such as; Age, sex, educational status, ability to perform any physical exercise, WHO stage at start of HAART drugs, general health conditions, length of time they stayed on ART treatment, comorbidity conditions with other diseases, having any clinical symptoms for HIV/AIDS, physical domain of quality of life, social domain of quality of life and the general quality of life status had statistically significant associations with HIV sero-positive status disclosure.

Similar to this study findings, the research done in China displayed that disclosing HIV sero- status to sexual partners was significantly related to better quality of relationship with partners and as well as open and effective communication within family members [26]. In similar to the above study, the other study done as Africa continent also shown that fear of discriminations; an anticipated disruption of relationships in their social life, a desire to protect oneself and others emotions possibility of verbal and physical abuse, stigma and confidentiality of their HIV positive sero - status information were factors that have statistical associations with HIV positive sero-status disclosure [18]. Likewise, the study done in South Africa showed that women were more likely to disclose their HIV positive sero - status as compared to their male counterparts [27].

In additional in concordant to this study findings, the study done in Mekelle Hospital, Ethiopia publicized that the knowledge of HIV status of their sexual partners, getting pretest counseling from caregivers, passing longer time since the HIV testing positivity detected, knowing people who disclose their HIV positive sero - status to the community before and having discussion with their sexual partners prior to HIV testing could influence disclosure of HIV status [22]. Likewise, this study results supported by the study done in Kemissie District, Northeast Ethiopia that shown prior discussions on HIV related issues, a smooth relationship with their sexual partners and communities and knowing the sexual partner status before were significantly associated with disclosure of their HIV positive sero-status [16]. The other factors that can affect HIV sero- status disclosure were also duration of remain on HIV related care follow up, Marital status, length of time since HIV test was done, knowing the partners HIV status before and prior discussion to each other with sexual partners were the main factors which affect the practice of HIV positive sero-status disclosure [7, 24, 25, 28].

### Strengths and limitations

The data of this study were collected by their caregivers and no discomforts were resulted related to their information secret and this can decrease the social desirability bias and fear of information leakage.. Since it’s done only in one hospital, generalizing of these findings for entire population as the region cannot possible. Moreover, the study participants were selected in a systematic random sampling method that can result may be homogeneous or repetition of study participants that had the similar characteristics by its nature. Also data were collected only by quantitative methods and it doesn’t address the information that possible to address only by qualitative methods. Therefore, in future, it is better if both qualitative and quantitative methods of data collections will considered while conduction of investigation on similar study title.

## Conclusions

The findings of this study indicated that the disclosure of HIV positive sero - status were very low. Age, sex, educational status, ability to perform any physical exercises, WHO stage at start of HAART drugs, general health conditions, length of time stayed on HAART treatment, presence of comorbidities, presence of any clinical symptoms for HIV/AIDS, physical domain of quality of life social domain of quality of life and the general or overall quality of life status were those factors that affected HIV sero-status positive disclosure.

## Abbreviations

AIDS: Acquired Immunodeficiency Syndrome
AOR: Adjusted Odds Ratio
ART: Antiretroviral therapy
CI: Confidence interval
COR: Crude Odds Ratio
HAART: Highly active antiretroviral therapy
HIV: Human Immune Deficiency Virus
JUSH: Jimma University Specialized Hospital
PLWHA: People Living with HIV/AIDS
WHO: World Health Organization
